# Recognizing opportunities when individual engaged in intrapreneurship: The role of creative self-efficacy and support for innovation

**DOI:** 10.3389/fpsyg.2022.937971

**Published:** 2022-07-26

**Authors:** Fangwei Liao, Anya Li, Qiang Zhang, Jin Yang

**Affiliations:** School of Economics and Management, Southwest University of Science and Technology, Mianyang, China

**Keywords:** intrapreneurship, opportunity recognition, creative self-efficacy, support for innovation, social cognitive theory

## Abstract

According to social cognitive theory, this study explored the relationship between intrapreneurship and opportunity recognition. We developed a moderated mediation model of creative self-efficacy as a mediator and support for innovation as a moderator linking intrapreneurship with opportunity recognition. Using a sample of 206 college students from Chinese universities, we found that intrapreneurship is positively related to opportunity recognition, and this relationship was mediated by creative self-efficacy. Our research further found that the effect of intrapreneurship on opportunity recognition was conditional on support for innovation. Finally, the theoretical and practical implications are discussed.

## Introduction

Intrapreneurship is the key to growth-hungry business organizations, an increasingly important tool for practitioners to improve corporate performance, an incubator for innovation within entrepreneurship and the effective tool for opportunity development (Rigtering and Weitzel, 2013; Azis and Amir, 2020). Therefore, it has become an important research area in the field of management research (Reuther et al., 2017; Blanka, 2019). Intrapreneurship which has adopted different definitions were based on various theoretical concepts and perspectives, and contributions to the field are also scattered (e.g., Amo, 2010; Antoncic and Antoncic, 2011; Turro et al., 2016). Prior research on intrapreneurship mainly focused at the organizational level (e.g., Åmo and Kolvereid, 2005; Gündoğdu, 2012; Dung and Giang, 2021; Abdelwahed et al., 2022). However, studies that explored it at the individual level remain scant (Gawke et al., 2017; Blanka, 2019). Consequently, we study the mechanism of intrapreneurship at the individual level, which is “the subjective motivation and expected behavior of individuals, which aims to create new business for the organization (i.e., venture behavior) and enhance the ability of the organization to respond to internal and market progress (i.e., strategic renewal behavior)” (Gawke et al., 2019, p. 815). The key behaviors of intrapreneurship at the individual level are individual initiative, active information search, thinking outside the box, speaking out, finding a way, and a degree of risk-taking (Wennekers and De Jong, 2008). Some scholars have proposed that the effective impact of intrapreneurship on organizations comes from the actions of individuals or employees themselves (Sinha, 2021). Therefore, this study aims to explore the impact mechanism of intrapreneurship on individual behaviors.

Opportunity recognition is a process by which individuals create and develop new businesses, markets, and technologies by recognizing and discovering potential opportunities (Shane and Venkataraman, 2000). That is, the process of recognizing opportunities is important not only for the new venture creation, but also for organizational strategy, adaptation, learning, and renewal (Grégoire et al., 2010; George et al., 2016). Therefore, exploring the relationship between intrapreneurship and opportunity recognition can play an important role for new venture creation and the future development trend of enterprises (Grégoire et al., 2010; Rigtering and Weitzel, 2013; George et al., 2016; Azis and Amir, 2020; Sinha, 2021). Individual participation in intrapreneurship to recognize opportunities is the first step for an organization to create performance and develop new venture strategies (Cherrington et al., 2021). However, the research of intrapreneurship on opportunity recognition has not been empirically studied (e.g., Covin and Miles, 1999; Thompson, 1999; Ireland et al., 2009; Sinha, 2021). To bridge this gap, we intend to investigate the effect of intrapreneurship on opportunity recognition, which would help expand the previous research and provide competitive advantage for enterprises.

However, relatively few studies actually analyzed how intrapreneurship is successful in opportunity recognition (Neessen et al., 2021), and the relationship is not firmly established. The basic assumption of our model is that individuals when engaging in intrapreneurship, influenced by underlying cognitive tendencies (Neessen et al., 2019; Cherrington et al., 2021), can be more successful in opportunity recognition by applying their own abilities from the current environment (Gawke et al., 2017; Neessen et al., 2021). Moreover, the effect of intrapreneurship on opportunity recognition can be explained by analyzing the links between psychological factors, environmental factors and individual behaviors (Wakkee et al., 2010; Blanka, 2019). Therefore, from the two aspects of internal psychological and external environmental factors, this paper intends to analyze why intrapreneurship can be successful in opportunity recognition (Neessen et al., 2021) *via* social cognitive theory (Bandura, 1997, 2012, 2018).

First is from the perspective of psychological mechanism. We offer a theoretical explanation that the link between intrapreneurship and opportunity recognition is mediated by creative self-efficacy. Recent studies have largely used psychological mechanisms to clarify the impact of intrapreneurship on individual behaviors (e.g., Gawke et al., 2017, 2018; Kim and Park, 2018; Blanka, 2019; Pandey et al., 2020). According to social cognitive theory, individuals tend to pursue their own goals if they believe that their abilities and actions can achieve the desired results (Bandura, 1999, 2012). Creative self-efficacy, which was defined as “the belief one has the ability to produce creative outcomes” (Tierney and Farmer, 2002, p. 1138), has been studied as a bridge linking individual activities to the process of opportunity recognition (e.g., Tumasjan and Braun, 2012; Koçak et al., 2013; Urban and Galawe, 2020). Nonetheless, some scholars have pointed out that previous literature highlighted the importance of creative self-efficacy in opportunity recognition (Gibbs, 2009; Tumasjan and Braun, 2012; Laguía et al., 2019). However, attention has scarcely looked into how existing sociocognitive variables (e.g., intrapreneurship; Hostager et al., 1998; Gibbs, 2009; Wakkee et al., 2010; Blanka, 2019) translate into opportunity recognition by developing creative self-efficacy (Gibbs, 2009; Muavia et al., 2022). Based on social cognitive theory (Bandura, 1999, 2012), the psychological cognitive process about intrapreneurship through creative self-efficacy is the key of opportunity recognition (Ciuchta and Finch, 2019; Camelo-Ordaz et al., 2020; Yasir et al., 2020). Individuals engaging in intrapreneurship can strengthen their subjective sense of mastery and confidence in producing creative results (Van-Brusel and Ulijn, 2008; Gawke et al., 2018). It will result in higher creative self-efficacy, and thus enhance individuals’ perseverance and motivation in the face of perceived challenge and uncertainty of opportunity (Gibbs, 2009; Michael et al., 2011; Tumasjan and Braun, 2012; Rigtering et al., 2019), which will lead to more success in opportunity recognition. Notwithstanding, at present, the psychological cognitive process behind individual intrapreneurship has not been deeply involved, and thus needs further research (Blanka, 2019; Yali and Changwei, 2021). This paper therefore responds to the overdue call made by Tumasjan and Braun’s (2012) and Yali and Changwei’s (2021) for further research aimed at exploring the cognitive processes behind intrapreneurial behavior by studying the mediating role of creative self-efficacy between intrapreneurship and opportunity recognition.

Second is the interaction between external environment and internal psychological factors. Intrapreneurial activities that individuals engaged in require a productive and inspiring environment (Blanka, 2019; Begeç and Arun, 2020). Therefore, intrapreneurship would be affected by the supportive environmental factors (Reuther et al., 2017; Blanka, 2019). Previous studies have analyzed the environmental factors that influence intrapreneurial activities (e.g., Antoncic and Hisrich, 2001; Hornsby et al., 2013). For example, Blanka (2019) proposed that individuals’ intrapreneurial behaviors would be affected by environmental factors, such as innovative workplace. Johnson and Wu (2012) investigated the impact of the interaction between job satisfaction and personal-environment fit on individuals’ participation in intrapreneurship. Support for innovation, as a supportive environment factor (Scott and Bruce, 1994; Jaiswal and Dhar, 2015), has been used by scholars to investigate its influence on an individual’s behavior (e.g., Jung et al., 2003; Dragoni, 2005; Gumusluoğlu and Ilsev, 2009; Chen et al., 2019; Akbari et al., 2020).

According to social cognitive theory (Bandura, 1999, 2012), individuals have the ability to influence their own behaviors through the interaction of cognitive, emotional and environmental factors (Bandura, 1997, 1999, 2012). Specifically, support for innovation contributes to the development and accumulation of individual cognition and social relationship (Jaiswal and Dhar, 2015; Çekmecelioğlu and Özbağ, 2016; Duan and Li, 2020) and can help individuals utilize and maintain their creative potential (Williams and Foti, 2011), which intensifies the influence of their creative self-efficacy on opportunity recognition (West, 1990; Bagheri, 2017). Moreover, support for innovation can provide psychological and physical resource to support individuals when they engaged in intrapreneurship (Gumusluoğlu and Ilsev, 2009; Hsiao et al., 2014; Ma and Corter, 2019). Accordingly, they can show stronger motivation, enthusiasm and belief to produce creative outcomes (Çekmecelioğlu and Özbağ, 2016; Duan and Li, 2020), which will contribute to enhanced social cognitive activities of opportunity recognition (Grégoire et al., 2010; George et al., 2016). Therefore, based on social cognitive theory (e.g., Bandura, 1997, 2012, 2018; Gibbs, 2009; Hornsby and Goldsby, 2009), we hypothesize that the extent to which intrapreneurship with opportunity recognition through creative self-efficacy may depend on the level of support for innovation.

**Figure 1:**
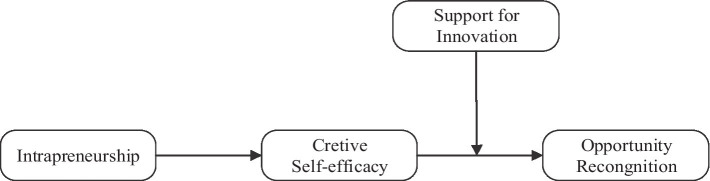
Conceptual model.

Overall, in order to reveal the specific mechanisms how do individuals recognize opportunities when they engage in intrapreneurial activities, we examined a moderated mediation model proposing creative self-efficacy as a mediator in the relationship between intrapreneurship and opportunity recognition, while the support for innovation perceived by individuals participating in intrapreneurship is proposed to moderate the second path from creative self-efficacy to opportunity recognition. Figure 1 shows the research model.

## Literature review and hypothesis

### Intrapreneurship and opportunity recognition

Intrapreneurship is “an individual agentic and anticipatory behaviors aimed at creating new businesses for the organization (i.e., venture behavior) and enhancing an organization’s ability to react to internal and external advancements (i.e., strategic renewal behavior)” (Gawke et al., 2019, p. 815). Intrapreneurship at the individual level is about bottom-up, proactive, and work-related initiatives of individuals (Blanka, 2019). Specifically, it involves innovative thinking, initiative, responsibility, advocacy, and some degree of risk-taking (Moriano et al., 2014). Its literatures have centered on innovation drive, the creation of new risks, the acquisition and utilization of external and internal sources of knowledge, and the development of new businesses (Gawke et al., 2017; Blanka, 2019; Begeç and Arun, 2020; Audretsch et al., 2021).

In particular, some studies have presented that intrapreneurship is an essential process for individuals to recognize opportunities and reallocate resources to take advantage of them (Rigtering and Weitzel, 2013; Sinha, 2021). Opportunities could be recognized (Short et al., 2010) as a mandatory prerequisite for the entire innovation process (Dayan et al., 2013; Rigtering et al., 2019). Likewise, recognizing and implementing new opportunities are a key prerequisite for the company to maintain outstanding performance (Wiethe-Körprich et al., 2017). Therefore, on the basis of social cognitive theory, this paper intends to explore the relationship between intrapreneurship and opportunity recognition.

This study proposes that intrapreneurship would improve opportunity recognition. First, when an individual engages in intrapreneurship, they would actively explore new business opportunities (Onyebu and Oluwafemi, 2018) and actively take actions on the opportunities found (McDowell, 2017). Concretely, intrapreneurship would improve individuals’ ability to perceive opportunities through tacit knowledge (Hayton and Kelley, 2006), abundant experiences (Gaertner, 2015), and existing resources (Smith et al., 2009). Opportunity recognition is a process of individual cognition (Grégoire et al., 2010). Social cognitive theory supposed that individuals are agents and active contributors to the development of their living environment (Bandura, 2018). One of the characteristics of intrapreneurship is individual initiative (Gawke et al., 2019), and when an individual engages in intrapreneurship, they will obtain the role recognition of “activities to recognize, explore and develop innovative opportunities through a systematic and collaborative approach” (Puech and Durand, 2017; Neessen et al., 2019; Begeç and Arun, 2020). Therefore, under the influence of latent cognitive tendency, individuals will actively seek and recognize opportunities when they engage in intrapreneurship (Ross, 1987; Eckhardt and Shane, 2003; Felício et al., 2012).

Second, social cognitive theory suggests that individual traits, thinking, abilities, and other psychosomatic functions influence and guide the generation of his or her behavior (Bandura, 1999). When individuals engage in intrapreneurship would use their creative thinking skills, a sensitivity to opportunities and domain-related skills (Moriano et al., 2014; Hador and Klein, 2019; Begeç and Arun, 2020), they will consciously capture specific information (Bosma et al., 2013; Rivera, 2017), and creatively use the information that is conducive to opportunity recognition (Wennekers and De Jong, 2008; Hsieh and Kelley, 2016). It will allow them to spontaneously recognize opportunities (Eckhardt and Shane, 2003). Although no empirical study exists on the positive impact between intrapreneurship and opportunity recognition, some studies can provide preliminary support for our research (Wennekers and De Jong, 2008; Antoncic and Antoncic, 2011; Baggen et al., 2016; Gawke et al., 2019). For instance, Heinze and Weber (2016) pointed out that individuals can create opportunities and simultaneously develop it during the intrapreneurship process (Onyebu and Oluwafemi, 2018; Blanka, 2019). Gawke et al. (2017) also showed that when individuals engage in intrapreneurship, their ability to seize opportunities will be improved through strategic renewal and risk-taking behaviors. Therefore, we propose the hypothesis:

*H1*: Intrapreneurship is positive related to opportunity recognition.

### Mediating role of creative self-efficacy between intrapreneurship and opportunity recognition

Creative self-efficacy is defined as “the belief one has the ability to produce creative outcomes” (Tierney and Farmer, 2002, p. 1138). Self-efficacy affects individual cognitive function, and may predict, regulate, and influence work behavior (Wood and Bandura, 1989; Pajares and Graham, 1999). It also tends to increase the level of individual effort and persistence (Tumasjan and Braun, 2012), which is essential for the successful generation of creative outcome (Puente-Díaz, 2016). In the previous literature, intrapreneurship is a collection of innovation input and positive reform behavior exhibited by individuals (Blanka, 2019; Li et al., 2021b), which can strengthen creative self-efficacy (Karwowski, 2014; Farmer and Tierney, 2017; Pandey et al., 2020) to realize innovation achievements (Gawke et al., 2018). Some scholars also suggested that individuals would be more willing to make efforts to sweat, and persevere until they reach their goals when faced with difficulties and challenges at high level creative self-efficacy (Richter et al., 2012; Farmer and Tierney, 2017), and they would show stronger motivation to seek opportunities (Ozgen, 2003; Yu, 2013; Li et al., 2021a). Empirically, creative self-efficacy is a powerful trigger of opportunity recognition (Yu, 2013), which is particularly suitable for the study of opportunity recognition (Gibbs, 2009; Tumasjan and Braun, 2012). Thus, we contend that creative self-efficacy bridges between intrapreneurship and opportunity recognition.

Intrapreneurship would improve creative self-efficacy through several ways. First, creative self-efficacy is derived partly from subjective feelings of mastery and confidence (Tierney and Farmer, 2002; Storme and Celik, 2018). For example, when individuals engage in intrapreneurship, they would show great enthusiasm for any risky and innovative behaviors (Pandey et al., 2020). Therefore, they would be more likely to perceive positive personal pursuits than perceived threats and expectations of failure (Douglas and Fitzsimmons, 2013; Gawke et al., 2019). Social cognitive theory suggests that an individual’s behavior affects his or her own cognition (Bandura, 1999). Therefore, intrapreneurship increases individuals’ cognition of their subjective sense of mastery and sense of confidence in producing creative outcomes (Van-Brusel and Ulijn, 2008; Gawke et al., 2018), which would further improve creative self-efficacy (Pandey et al., 2020).

Second, another core factor in establishing creative self-efficacy (Bandura, 1997) is the positive feedback of past creative performance and achievement (Li et al., 2021a). For example, when individuals engage in intrapreneurship, he or she will receive positive feedback on the outcomes of innovation (Gawke et al., 2017). In addition, they would gain new knowledge, experience (Honig, 2001), enhanced creative thinking skills (Pandey et al., 2020), and the development of self-career (Van-Brusel and Ulijn, 2008). They also expect similar positive experiences when performing such behaviors (Bandura, 1997; Marvel et al., 2007). After receiving such positive feedback, individuals’ expectations and beliefs of achieving creative results would increase (Pinchot and Pellman, 1999; Karwowski, 2014; Pandey et al., 2020). Social cognitive theory also suggests that helping individuals overcome anxiety and fear (Ng and Lucianetti, 2016) could contribute to the establishment of creative self-efficacy (Bandura, 1999). Intrapreneurship would help individuals overcome their fears and difficulties in achieving their goals (Anderson and Jack, 2002; Wennekers and De Jong, 2008; Rigtering and Weitzel, 2013), helping them become more optimistic and hopeful (Van den Heuvel et al., 2015). Therefore, individuals can develop their creative self-efficacy by obtaining positive emotional feedback on their innovation achievements when they engage in intrapreneurship (Tierney and Farmer, 2002; Tumasjan and Braun, 2012; Gawke et al., 2017). Empirical evidence in the existing literature of intrapreneurship confirms the positive effect of intrapreneurship on creative self-efficacy (e.g., Di Fabio, 2014; Pandey et al., 2020).

Creative self-efficacy is an effective predictor of opportunity recognition (Tumasjan and Braun, 2012). First, individual with creative self-efficacy tend to show stronger motivation to recognize opportunities (Tierney and Farmer, 2002; Pech and Cameron, 2006). Specifically, in any given situation, creative self-efficacy makes individuals optimistic and enhances inclination of individuals to focus on pursuing potentially valuable opportunities (Richter et al., 2012; Koçak et al., 2013). Social cognitive theory suggests that a personal motivation to perform a particular activity or task is dependent on the individual’s judgment of his or her abilities and expectations about activities’ outcomes (Bandura, 1997, 1999; Slåtten, 2014). Creative self-efficacy can improve the endeavor and persistence level of individuals (Cai et al., 2019), which will increase their inclination to recognize and believe the positive results achieved by using their own creative thinking and ability (Bandura, 2012; Yu et al., 2019). Therefore, creative self-efficacy enhances individuals’ motivation, expected judgment, and cognition when they participate in opportunity recognition (Ozgen, 2003; Li et al., 2005; Tumasjan and Braun, 2012; Slåtten, 2014; Afriyie, 2020).

Second, strong creative self-efficacy can broadly motivate individuals to seek advice and guidance in the application of creative behaviors (Richter et al., 2012) where they will feel confident about their knowledge and skills, thus it would generate ideas and implement them in their work (Jiang and Gu, 2017). It will also influence them to become more confident about the success of their creative efforts (Ozgen and Baron, 2007; Tierney and Farmer, 2011; Cai et al., 2019). It can promote individuals to recognize opportunities (Tumasjan and Braun, 2012; Rigtering et al., 2019) through creative cognitive processes (Michael et al., 2011). Empirical evidence has confirmed that creative self-efficacy positively can affect opportunity recognition (e.g., Gibbs, 2009; Tumasjan and Braun, 2012; Baggen et al., 2016).

To sum up, intrapreneurship forms a cognitive framework that influences opportunity recognition through creative self-efficacy. Specifically, intrapreneurship can develop individuals’ ability and confidence in creative problem solving (Gawke et al., 2018; Pandey et al., 2020). They spend extra time on creative cognition, and have better confidence to take risks and perform creative actions (Jiang and Gu, 2017; Mehmood et al., 2020), thus enhancing creative self-efficacy. After individuals engaged in intrapreneurship demonstrated more creative self-efficacy, they encourage self-motivation, which leads to a more proactive search for information about opportunities (Gibbs, 2009) and more insightful recognition of opportunities in their current environment (Yasir et al., 2020). Therefore, we anticipate that creative self-efficacy bridges intrapreneurship and opportunity recognition. The following hypothesis is proposed:

*H2*: Creative self-efficacy mediates the positive relationship between intrapreneurship and opportunity recognition.

### Moderating role of support for innovation

Support for innovation is “the expectation, approval, and practical support of attempts to introduce new and improved ways of doing in the work environment.” (West, 1990, p. 38). Support for innovation not only provides freedom, social, and emotional support for individuals, but also provides material assistance, additional funds or work equipment and other resources (Ren and Zhang, 2015). As an important environmental factor, support for innovation can be reflected in perceptions of task and resource-related creative problems about solving support, perceptions (e.g., attitudes about revolution and innovation) and emotions (Scott and Bruce, 1994; Liu and Chan, 2017). Previous studies have examined the moderating effect of support for innovation. For example, stress and innovative performance (Leung et al., 2011), transformational leadership and organizational innovation (Gumusluoğlu and Ilsev, 2009; Mokhber et al., 2018), and organization’s ethical climate and innovation (Choi et al., 2013).

Nonetheless, previous studies have shown that creative self-efficacy can positively affect opportunity recognition (Gibbs, 2009; Tumasjan and Braun, 2012). However, creative self-efficacy varies by individual, and they might have different belief efficacies for the future due to distinct work environments and situations (Bandura, 2012; Li et al., 2021a). Individuals’ own cognition to affect their behaviors can be dynamically adjusted when they are affected by the environment (Bandura, 1997). Based on social cognitive theory (Bandura, 1999), a collaborative environment that promotes mutual help, support and coordination among individuals in innovation attempts (Liu and Chan, 2017) will motivate individuals to adhere to the confidence of opportunity recognition (West, 1990; Bagheri, 2017). Support for innovation can help individuals utilize and maintain their creative potential (Williams and Foti, 2011), which can intensify the influence of their creative self-efficacy on developing cognition and information for solving creative problems (Gong et al., 2009; Leung et al., 2011; Akbari et al., 2020). Thus, we suggest that support for innovation may be an important moderation mechanism for the relationship between creative self-efficacy and opportunity recognition.

First, individuals with high support for innovation will be encouraged to take initiative and risks, and will also be challenged to find innovative approaches to their work (Hornsby and Goldsby, 2009). This perceived support not only stimulates positive emotions, but also influences individuals to actively participate in the creative process more (Gilson and Shalley, 2004). According to social cognitive theory (Bandura, 1999, 2012), the external environment can shape the individual’s psychological cognition of determining and utilizing opportunities (Jiang et al., 2019) and arouses the individual’s positive emotions (Wang et al., 2018). Specifically, the creation of this supportive environment will provide protection for individuals (McDowell, 2017). And in this protective environment, they will be driven to take risks, think, and act innovatively (Scott and Bruce, 1994; Amabile et al., 1996; Bagheri, 2017). Therefore, individuals with high support for innovation will maintain a stable level of innovative positive emotion (Gawke et al., 2017, 2018), take advantage of opportunities created (Hsiao et al., 2014), and reinforce creative cognitive flexibility (Tajpour and Hosseini, 2019). Consequently, the beneficial effects of creative self-efficacy on opportunity recognition will increase.

Second, individuals with high support for innovation would realize that they have sufficient resources to support them (Scott and Bruce, 1994; Gumusluoğlu and Ilsev, 2009). Drawing from social cognitive perspective, creative self-efficacy can be gradually accumulated through the growth and development of individuals’ cognition and social relationships (Bandura, 1999; Pandey et al., 2020). Specifically, with this support and encouragement, individuals will share their knowledge of practices, procedures, policies, and ways among themselves (Çekmecelioğlu and Özbağ, 2016). Consequently, they experience greater freedom, confidence in their ability, and a sense of contribution (Çekmecelioğlu and Özbağ, 2016). Moreover, individuals with high support for innovation are given opportunities and support to develop their abilities (Hornsby and Goldsby, 2009), which can encourage them to open up, use creative suggestions, adopt innovative thinking, and take risks (Howell and Avolio, 1993). Therefore, individuals will show dedication and enthusiasm in their creative behavior (Bandura, 1999; Schaufeli and Bakker, 2004; Puente-Díaz, 2016). Accordingly, it increases the likelihood of creating and recognizing opportunities (Ford, 1996; Puente-Díaz, 2016; Kim et al., 2018). In this supportive environment, the positive impact of creative self-efficacy on opportunity recognition will also be enhanced. Consequently, support for innovation provides emotional and physical support for individuals (Hornsby and Goldsby, 2009), which strengthens the influence of creative self-efficacy on opportunity recognition. Thereby, the following hypothesis is proposed:

*H3*: The relationship between creative self-efficacy and opportunity recognition is moderated by support for innovation, and the relationship is stronger when support for innovation is high.

Assuming that support for innovation moderates the positive impact of creative self-efficacy on opportunity recognition, then a supportive environment perceived by individuals may conditionally affect the strength of the indirect relationship between intrapreneurship and opportunity recognition. Namely, the effect of belief and efficacy of the ability to solve creative problems gained by individuals engaged in intrapreneurship on opportunity recognition may be moderated by support for innovation, thus demonstrating a moderated mediation effect. A strong positive association between creative self-efficacy and opportunity recognition when support for innovation is high, as we assume, then support for innovation will positively moderate the mediation effect. That is, the mediation effect of creative self-efficacy on intrapreneurship and opportunity recognition will be stronger when support for innovation at a high level, as claimed in the following hypothesis:

*H4*: Support for innovation moderates the indirect effect of intrapreneurship on opportunity recognition (*via* creative self-efficacy). Specifically, creative self-efficacy positively mediates the indirect effect when support for innovation is high.

## Materials and methods

### Sample and procedures

In this study, convenient sampling method was adopted, and the sample objects were selected from the entrepreneurial teams among college students and their team members participating in the Sichuan Provincial Innovation and Entrepreneurship Competition in Southwest China. The research team contacted the leaders of the participating teams, proposed research objectives, and ensured the confidentiality of the responses. Students from 69 teams were invited to participate. Paper questionnaires to those team leaders and team members were distributed by researchers, and questionnaires were collected at the site. Participants were informed of the purpose of the survey and the procedures for filling out the questionnaire, and all information they provided was guaranteed confidentiality. A total of 281 questionnaires were sent out. Finally, 206 usable questionnaires were selected for this study with a response rate of 73.31%.

Slightly over half (64.10%) of the participants were women, the vast majority of them (89.80%) were between the ages of 20 and 22, and most have academic talent (66.50%). The demographic profile of participants is presented in Table 1.

**Table 1:** Demographic profile of participants.

Item	Category	Frequency	%
Gender	Male	74	35.90
	Female	132	64.10
Age	20 years old or less	89	43.20
	21 years old	68	33.00
	22 years old	28	13.60
	23 years old	9	4.40
	24 years old	8	3.90
	25 years old	2	1.00
	26 years old	2	1.00
Professional category	Academic	137	66.50
	Professional	69	33.50

*N* = 206.

### Measures

To ensure the effectiveness in this survey, the measurement methods used in this survey were adapted from existing literature. Translation and back-translation was performed to ensure the questionnaires’ consistency (Brislin, 1970). The survey used a five-point Likert scale (1 = never, 5 = very frequently).

### Intrapreneurship

Intrapreneurship was measured using a three-item scale from Moriano et al. (2014). Sample items included “I take the initiative to start projects,” “I take calculated risks despite the possibility of failure,” and “I develop new processes, services or products.” The Cronbach’s alpha for this measure was 0.73. We averaged all the 3-item to create an overall intrapreneurship score.

### Creative self-efficacy

Creative self-efficacy was measured using a 3-item scale proposed by Tierney and Farmer (2002). Sample items included “I have confidence in my ability to solve problems creatively,” “When facing difficult tasks, I am certain I will accomplish them creatively,” and “I feel that I am good at generating novel ideas.” The Cronbach’s alpha for this measure was 0.80. All the three items were averaged to create an overall creative self-efficacy score.

### Support for innovation

Support for innovation was measured using an 8-item scale proposed by Anderson and West (1998). Three examples of these questions were the following: “The level of commitment to pursuing innovative working methods in the job,” “The time guaranteed by the company for innovation,” and “Team members have a lot to gain and pay for innovation.” The Cronbach’s alpha for this measure was 0.91. We averaged all the 8 items to create an overall support for innovation score.

### Opportunity recognition

Opportunity recognition was measured using a 3-item scale proposed by Ozgen and Baron (2007). Three examples of these questions were the following: “The level of commitment to pursuing innovative working methods in the job,” “The time guaranteed by the company for innovation,” and “Team members have a lot to gain and pay for innovation.” The Cronbach’s alpha for this measure was 0.86. We averaged all the 3 items to create an overall opportunity recognition score.

### Control variables

We controlled for three demographic variables, age (1, “male,” 0, “female”), gender (in years), and the student’s major category (1, “academic,” 0, “professional”) given their significant effect on opportunity recognition found in previous studies (e.g., Davidsson and Honig, 2003; Arenius and Clercq, 2005; DeTienne and Chandler, 2007; Dahalan et al., 2013; Hannibal et al., 2016). In addition, age also influences an individual’s intention to engage in intrapreneurship (Hador and Klein, 2019). Thereby, we controlled for these factors in the following analysis.

## Analyses and result

### Reliability and validity

Confirmatory factor analysis was conducted using SPSS 24.0 and AMOS 23.0 to assess the reliability and validity of the scale. Content validity, convergent validity and discriminant validity were assessed in our analysis. The questionnaire items were in line with the extant literature; thus, the content validity was evaluated. Table 2 shows that Cronbach’s alpha ranged from 0.78 to 0.91, indicating that all variables have acceptable reliability. All items’ factor loadings are higher than the 0.70 criterion. Table 2 also shows that the composite reliability ranged from 0.87 and 0.93, higher than the recommended level of 0.70. All construct’s average variance extracted (AVE) scores are higher than 0.5 which ranged from 0.69 and 0.73. These results demonstrate that we have good convergent validity (Fornell and Larcker, 1981). The relationship between constructs and the square root of AVE score was also compared to evaluate the discriminant validity of the project. Table 3 displays that the square root of AVE score of each construct is greater than the correlation between constructs, thus confirming the discriminant validity of this construct.

**Table 2:** Factor loadings, Cronbach’s alpha (*α*), composite reliability, and average variance extracted (AVE).

No	Variables	Loading	Cronbach’s *α*	Composite reliability	AVE
1	Intra	0.81–0.88	0.78	0.87	0.70
2	Cse	0.83–0.87	0.80	0.89	0.72
3	Opp	0.81–0.87	0.86	0.89	0.73
4	Ia	0.78–0.89	0.91	0.93	0.69

*N* = 206.

Intra, intrapreneurship, Cse, creative self-efficacy, Opp, opportunity recognition, Ia, support for innovation.

**Table 3:** Means, standard deviations, correlations, and square roots of AVE in diagonals.

No	Variables	Mean	SD	Gender	Age	Categ	Intra	Cse	Opp	Ia
1	Gender	1.64	0.48							
2	Age	2.00	1.22	−0.09						
3	Categ	1.33	0.47	−0.05	−0.14^*^					
4	Intra	3.79	0.70	0.02	−0.03	−0.18^*^	(0.84)			
5	Cse	3.64	0.66	−0.07	0.02	0.01	0.30^**^	(0.85)		
6	Opp	3.68	0.74	0.00	0.04	−0.08	0.30^**^	0.37^**^	(0.86)	
7	Ia	3.46	0.66	−0.04	0.11	0.00	0.09	0.51^**^	0.32^**^	(0.83)

* *p* < 0.05;

** *p* < 0.01;

*** *p* < 0.001.

*N* = 206.

Intra, intrapreneurship, Cse, creative self-efficacy, Categ, professional category, Opp, opportunity recognition, Ia, support for innovation. Values in parentheses on the diagonal are the square roots of AVE of each scale. Unadjusted correlations appear below the diagonal; Alpha coefficients are on the diagonal, in parentheses.

In addition, fit indices of the root mean square error of approximation (RMSEA), comparative fit index (CFI), Tucker–Lewis index (TLI) and chi-square statistics were also used to test the consistency of the study variables (Bandalos, 2002). The results from Table 4 showed that the fitting degree of our hypothesis model (Model 1) is better than other alternative models (*χ*^2^ = 181.63; *df* = 83; *χ*^2^/*df* = 2.19; CFI = 0.94; RMSEA = 0.07; TLI = 0.92). Therefore, the fit indices of Table 4 demonstrate the convergent and discriminant validity of the constructs studied.

**Table 4:** Results of confirmatory factor analyses.

No	Models	*χ* ^2^	*df*	*χ*^2^/df	CFI	TLI	RMSEA
1	4-Factor Model	181.63	83	2.19	0.94	0.92	0.07
2	3-Factor Model (inta + cse, opp, ia)	336.08	87	4.21	0.83	0.79	0.13
3	2-Factor Model (inta + cse, opp + ia)	572.75	89	6.44	0.70	0.65	0.16
4	1 Factor Model	735.23	90	8.17	0.60	0.53	0.19

*N* = 206.

Intra, intrapreneurship, Cse, creative self-efficacy, Opp, opportunity recognition, Ia, support for innovation.

### Common method bias

Based on Harman’s one-factor test (Podsakoff and Organ, 1986), results show that four factors that account for 74.58% of variance are extracted and the first factor accounts for 36.67%. Thus, although the data were collected from the same source, common method bias is not a major contaminant for our results.

### Descriptive statistics

Table 3 displays the means, standard deviations, correlations of the variables and square Roots of AVE. Intrapreneurship has positive influence on creative self-efficacy and opportunity recognition (*r* = 0.30, *p* < 0.01; *r* = 0.30, *p* < 0.01). In addition, creative self-efficacy has positive influence on opportunity recognition (*r* = 0.37, *p* < 0.01). These results are consistent with and provide preliminary support for our hypothesis. To solve multicollinearity, the variance inflation factor (VIF) of each regression equation was calculated. The maximum VIF is less than 1.09, well below the threshold of 5.00 or 10.00 (O’brien, 2007), which means that multicollinearity problems are minimal in the present research.

### Hypothesis testing

Linear regression and hierarchical multiple regression analysis were performed on Hypothesis 1 and 2. First, we choose age, gender, and professional category as control variables (Model 1 and 3). Then, linear regression was applied to explore the correlation between the independent variables (intrapreneurship), mediating variable (creative self-efficacy) and the dependent variables (opportunity recognition; Model 2, Model 4 and Model 5). Third, mediating variables were included in the regression analysis of independent variables to dependent variables (Model 6). Table 5 presents the results.

**Table 5:** Results of the mediating effects of creative self-efficacy.

	Creative self-efficacy		Opportunity recognition
	Model 1	Model 2	Model 3	Model 4	Model 5	Model 6
1. Gender	−0.07	−0.08	0.00	0.00	0.04	0.02
2. Age	0.02	0.03	−0.08	0.04	0.01	0.03
3. Categ	0.01	0.07	0.02	−0.02	−0.11	−0.04
4. Intra		0.30^***^		0.30^***^		0.20^*^
5. Cse					0.37^***^	0.31^***^
*R* ^2^	0.01	0.09	0.01	0.09	0.14	0.18
Δ*R*^2^	0.01	0.08	0.01	0.08	0.05	0.04
*F*	0.38	5.22^**^	0.47	5.05^***^	8.44^***^	8.75^***^

* *p* < 0.05;

** *p* < 0.01;

*** *p* < 0.001.

*N* = 206.

Intra, intrapreneurship, Cse, creative self-efficacy, Categ, professional category.

Table 5 shows that intrapreneurship is significantly related to creative self-efficacy (*β* = 0.30, *p* < 0.001, Model 2) and opportunity recognition (*β* = 0.30, *p* < 0.001, Model 4). Moreover, creative self-efficacy is significantly associated with opportunity recognition (*β* = 0.37, *p* < 0.001, Model 5). Third, the effect of intrapreneurship on opportunity recognition (*β* = 0.20, *p* < 0.05, Model 6) is significant when creative self-efficacy is included in the regression equation, and creative self-efficacy remains significantly related to opportunity recognition (*β* = 0.31, *p* < 0.001, Model 6), indicating that the relationship between intrapreneurship and opportunity recognition is not fully mediated by creative self-efficacy. Thus, Hypothesis 1 and 2 are supported.

The bias-corrected bootstrapping procedure developed by Preacher and Hayes (2008) was also used to further test Hypotheses 2.

Table 6 shows that the indirect effect of intrapreneurship on opportunity recognition *via* creative self-efficacy is positive and significant (indirect effect = 0.090, 95% CI = 0.04–0.16), which is excluded zero. Thus, Hypotheses 2 is supported. The model is significant, adjusted *R*^2^ = 0.18, *F* (5, 200) = 8.75, *p* < 0.001.

**Table 6:** Indirect effects of intrapreneurship (*via* creative self-efficacy) on opportunity recognition.

Path	Intrapreneurship → creative self-efficacy → opportunity recognition
Bootstrap-indirect effect	0.08
Lower limit 95% *CI*	0.04
Upper limit 95% *CI*	0.16

^*^p < 0.05;

^**^p < 0.01;

^***^p < 0.001.

*N* = 206.

Adjusted *R*^2^ = 0.18, *F* (5,200) = 8.75, *p* < 0.001. Confidence intervals are bias-corrected based on 1,000 bootstrap samples. Control variables: gender, age, professional category.

In this study, hierarchical adjustment regression analysis was used to test Hypothesis 3. Control variables are entered in Step 1; the independent variable is entered in Step 2; the moderator is entered in Step 3; finally, the interaction term is entered. To avoid multicollinearity, independent (creative self-efficacy) and moderator (support for innovation) variables are centered in the regression analyses (Aiken et al., 1991).

**Figure 2:**
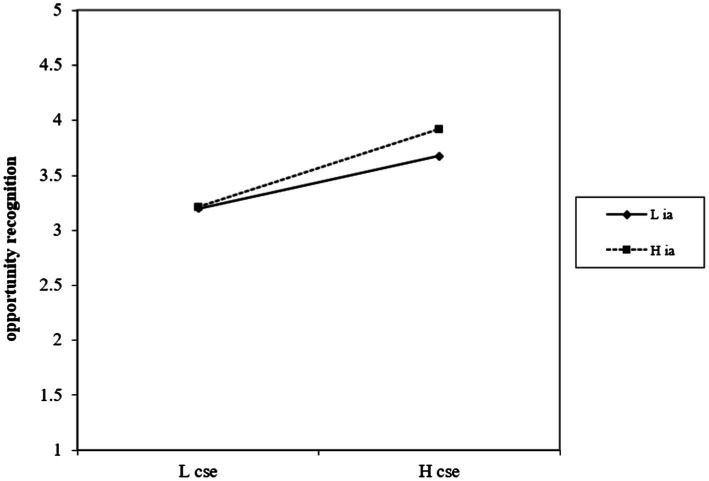
Plot of interaction between creative self-efficacy (cse) and opportunity recognition. High support for innovation (ia) is indicated by a square; low support for innovation (ia) is indicated by a diamond.

As shown in Table 7, the interaction between creative self-efficacy and support for innovation is positively related to opportunity recognition (*β* = 0.25, *p* < 0.001, Model 4). Figure 2 shows creative self-efficacy is more positively related to opportunity recognition at the high-level of support for innovation. Consistent with our hypotheses, results show that support for innovation positively moderates the direct relationship between creative self-efficacy with opportunity recognition. Accordingly, Hypothesis 3 is supported.

**Table 7:** Results of the moderating effects of support for innovation.

	Opportunity recognition			
	Model 1	Model 2	Model 3	Model 4
Gender	0.00	0.02	0.03	0.02
Age	0.02	0.02	0.00	0.01
Categ	−0.08	−0.08	−0.08	−0.05
Cse		0.37^***^	0.28^***^	0.36^***^
*Ia*			0.17^*^	0.19^**^
Cse × *Ia*				0.25^***^
*R* ^2^	0.08	0.14	0.22	0.25
Δ*R*^2^	0.08	0.06	0.08	0.03
*F*	0.47	8.44^***^	7.97^***^	9.39^***^

* *p* < 0.05;

** *p* < 0.01;

*** *p* < 0.001.

*N* = 206.

Intra, intrapreneurship, Cse, creative self-efficacy, Categ, professional category, Ia, support for innovation.

Hypothesis 4 predicts that support for innovation moderates the intrapreneurship—creative self-efficacy—opportunity recognition mediating linkage. To test for moderated mediation, a regression-based approach was used to estimate the conditional indirect effects of the moderators (Preacher et al., 2007).

Table 8 shows that the conditional indirect effect for intrapreneurship on opportunity recognition was not significant when support for innovation was low (conditional indirect effect = 0.04, SE = 0.22, 95% CI = −0.01 to 0.09). Contrarily, when support for innovation was high (conditional indirect effect = 0.14, SE = 0.50, 95% CI = 0.05–0.26), it is significant. Thus, Hypotheses 4 is supported.

**Table 8:** Moderated mediation results for intrapreneurship across levels of support for innovation for opportunity recognition.

	Intrapreneurship		
Support for innovation on opportunity recognition	Conditional indirect effect	Boot SE	95% Bias-corrected bootstrap confidence interval
Low Ia	0.04	0.22	[−0.01,0.09]
High Ia	0.14	0.50	[0.05,0.26]

*N* = 206.

Ia, support for innovation.

## Discussion

First, by utilizing social cognitive theory (Bandura, 1997), our study aimed to understand the relationship between intrapreneurship and opportunity recognition. Then, we examined how intrapreneurship affects opportunity recognition through the cognitive process of creative self-efficacy. In addition, we discovered that creative self-efficacy can partially mediate the relationship between intrapreneurship and opportunity recognition. Our findings empirically support the argument that other mediating mechanisms should exist in the relation between intrapreneurship and individual outcomes (Blanka, 2019; Sinha, 2021).

Moreover, the cognitive process of opportunity recognition may be affected by supportive situations. This study examined support for innovation as a moderator to explore the relationship between creative self-efficacy and opportunity recognition. The evidence indicated that support for innovation can enhance the positive impact of creative self-efficacy on opportunity recognition. This finding is consistent with the viewpoint that when individuals perceive support for innovation, their belief and initiative to engage in creative activities are enhanced (Navaresse et al., 2014; Lukes and Stephan, 2017; Duan and Li, 2020). Moreover, we found that when individuals receive high psychological and physical support, the indirect relationship between intrapreneurship and opportunity recognition through creative self-efficacy is more significant. Thus, these findings suggest that individuals engaging in intrapreneurship can have some effects on opportunity recognition when they receive support for innovation and have the belief to complete the innovative activity.

### Theoretical implications

Our study examined the relationship of intrapreneurship on opportunity recognition and then provided some theoretical contributions. First, this current study resolved several theoretical gaps in the intrapreneurship literature. Previous studies about intrapreneurship have focused on investigating organizational level outcomes, such as growth (Antoncic and Antoncic, 2011) and performance (Ibrahim et al., 2016). However, research on individual outcomes of behavioral aspects of intrapreneurship remains scant (Reuther et al., 2017). To the best of our knowledge, this research is the first to empirically explore the impact of intrapreneurship on opportunity recognition, which has expanded the theoretical research of Ireland et al. (2009). Concurrently, we provide a theoretical framework to explain how individuals can enhance their innovation implementation behaviors (i.e., opportunity recognition) through intrapreneurship. We extend the research of Gibbs (2009) and Terán-Yépez and Guerrero-Mora (2020) on social cognitive models of opportunity recognition. We connect the “psychological” and “social” characteristics of individuals participating in intrapreneurship, and enrich the understanding of the psychological determinants of intrapreneurship (Chouchane et al., 2021). We also confirm the view of Begeç and Arun (2020) from following side, that is, when individuals participate in intrapreneurship, they will apply their own abilities to the existing environment, which will lead to recognition of their perceived opportunities. Our research that responds by increasing our understanding of the impact and process of intrapreneurship at the individual level contributes to the field of intrapreneurship research (Gawke et al., 2017; Blanka, 2019).

Second, our study responds to the call for investigation of the underlying psychological mechanisms between intrapreneurship and individual outcomes (Blanka, 2019; Pandey et al., 2020). On the basis of the previous significant contribution of Gawke et al. (2017) and Pandey et al. (2020), we extend the mediating mechanism of psychological mechanisms between intrapreneurship and individual outcomes. By establishing the role of creative self-efficacy as a mediator in the relationship between intrapreneurship and opportunity recognition, this current research identified the social cognitive process about intrapreneurship at the individual level. Meanwhile, our findings enrich the intrapreneurship literature by examining the mediating variables in individuals participating in intrapreneurship (Gawke et al., 2017). In addition, our research discussed the influence of intrapreneurship on the psychological mechanism of creative self-efficacy (Pandey et al., 2020), which contributes to creative self-efficacy literature by identifying intrapreneurship as its predictor. Our findings also enrich the relationship between creative self-efficacy and opportunity recognition of existing researches (Tumasjan and Braun, 2012; Baggen et al., 2016).

Third, our research supports the following viewpoint that support for innovation needed to interact with other innovation-related factors to influence individual creative expression (Gumusluoğlu and Ilsev, 2009; Lukes and Stephan, 2017), as a contextual factor. This study reveals that the indirect relationship between intrapreneurship and opportunity recognition through creative self-efficacy was conditional on support for innovation. Specifically, creative self-efficacy has a stronger influence on opportunity recognition when support for innovation is at a high level. Thus, our study examined the mechanism of innovation support as a moderating variable, enriching the growing body of research on innovation support (Gumusluoğlu and Ilsev, 2009). In addition, our research found that when individuals perceive that physical and psychological resources are supported, their belief in the ability to produce creative outcomes would be enhanced, which will transform into innovative outputs. This finding supports the argument that when individuals perceive support for innovation, it not only can trigger his or her positive emotions but also translate into more active participation in the creative process (Gilson and Shalley, 2004; Jin and Zhong, 2014; Kibirango et al., 2017). Ultimately, our study extends insights into creative self-efficacy in the work context (Puente-Díaz, 2016).

Fourth, the conceptual model designed in our research also had some theoretical contributions. The study aimed at evaluating a moderated mediation model to explore the indirect effect of intrapreneurship on opportunity recognition through creative self-efficacy moderated by support for innovation. This study investigated the process of intrapreneurship at the individual level based on the social cognition perspective. Our finding demonstrated that the intensity of the indirect relationship between intrapreneurship and opportunity recognition through creative self-efficacy was contingent on support for innovation. The current research supported that the impact of intrapreneurship on individual outcomes through individual cognitive mechanism was influenced by perceived work environment and support (Hornsby and Goldsby, 2009; Wakkee et al., 2010; Gawke et al., 2018; Begeç and Arun, 2020). Our study has enriched the research of individual intrapreneurship process under the framework of social cognition. Moreover, our study also responds to the suggestion of Begeç and Arun (2020) that the underlying psychological processes that environmental factors trigger and lead to subsequent changes in intrapreneurial behavior need specific analysis.

Last but not the least, compared with western countries, the study of intrapreneurship started late and is mostly discussed with concepts in China (Yali and Changwei, 2021). However, China’s economic reform and the participation of foreign companies in China’s economy have led to the popularization of Western-style management (Sun and Pan, 2011). Therefore, our research helps expand the study of intrapreneurship in the Chinese context. From the perspective of psychological cognition (Yali and Changwei, 2021), it provides a useful reference for promoting the integration of Chinese domestic research with international research.

### Practical implications

Our findings provide several practical implications for organizational managers as well as decision makers. First, these results suggest that promoting individual intrapreneurial activities in the workplace would entail a win–win situation operating in current commercial environments for organizations and their members (Bennett and Lemoine, 2014). Therefore, intrapreneurship can develop the human capital of an enterprise to adapt to future requirements (Pandey et al., 2020). Supporting individual intrapreneurship in the organization has been proven to increase individuals’ innovation output (Marques et al., 2021) and when individuals engage in intrapreneurship are self-motivated, enthusiastic, and innovative (Moriano et al., 2014). Consequently, managers should encourage individuals to participate in intrapreneurial activities. Example include carrying out education and training to promote intrapreneurship, filling them with curiosity and confidence about their career (Woo, 2018), and improving their initiative and skills in developing new projects (Chouchane et al., 2021).

Moreover, after intrapreneurship is successful in opportunity recognition, the next stage is for employee to engage in intrapreneurship to achieve growth and development for organizations through explicit capabilities (Cherrington et al., 2021). Therefore, managers can promote employees’ intrapreneurial behaviors through coaching (Wakkee et al., 2010), including developing new ideas to create performance for the enterprise by helping employees to gain access to other resources and expertise (Blanka, 2019). Additionally, more cross-border knowledge can be transferred to employees through mentoring, thus contributing to recognize opportunities (Yali and Changwei, 2021).Second, we provide insights into how intrapreneurship can facilitate individual participation in opportunity recognition through creative self-efficacy. Decision makers should take steps to promote the creative self-efficacy of organizational members. Creative self-efficacy is dynamic, which can actively promote individuals to participate in creative activities and take incentives for their failure or successful activity experiences (Liu et al., 2016). Moreover, managers can enhance supportive and non-controlling management styles, and provide care and trust in organization members to encourage them to develop new skills. Decision makers should also pay special attention to human-resource management activities, especially those related to organization members selection and executive empowerment, which can improve their autonomy and belief in creative activities. Employees who are empowered to use self-perception and validation skills to complete tasks are more likely to be successful in opportunity recognition (Tumasjan and Braun, 2012; Teng et al., 2020).

Finally, our research argues that support for innovation is an appropriate work environment that effectively promotes organization members’ beliefs and motivations to engage in creative behaviors. Thus, organizations can establish an open and supportive climate that can enhance individuals’ ability to develop new ideas and different solutions to problems (Akbari et al., 2020). An enabling environment allows employees to track and respond to customers’ needs and preferences proactively and flexibly, thus cultivating and promoting employees’ initiative in internal entrepreneurship (Sun and Pan, 2011). Managers can also improve a range of policies and institutions, allowing to enable individuals to properly face challenges from the environment and make them feel motivated and committed to the opportunity recognition process. In addition, managers can create a climate in which individuals are perceived to support for innovation by encouraging, approving, and rewarding their creative behavior and providing them with adequate resources, such as manpower, money, time (Gumusluoğlu and Ilsev, 2009), specific training, and development projects. Training and developing projects can help individuals gain opportunities to recognize relevant skills (McDowell, 2017) and intrinsic motivation (Farnese and Livi, 2016) when they participate in intrapreneurial activities. Managers should also expose members to the accumulated experience of projects and the enthusiastic expectations of the organization, thereby unleashing the initiative and ingenuity of intrapreneurship (Begeç and Arun, 2020).

### Limitations and directions for future research

Despite the merits of this study, we identified some limitations requiring attention and directions for future research. First, our ability to make definitive inferences to causality is limited by cross-sectional design. The significant relationship that could reveal the correlation in our study cannot reveal causation. For instance, the positive effects of individual engage in intrapreneurship on psychological capital, such as creative self-efficacy, will become increasingly complex over time (Gawke et al., 2017). The ability of individuals to recognize opportunities also strengthens the motivation of individuals to engage in intrapreneurship (Turro et al., 2016), which may be followed by intrapreneurship behaviors. Hence, future research could explain the significant relationship through a longitudinal study.

Second, we take entrepreneurial teams and individuals of college students in China as the research object. Although confirmatory factor analyses were performed, a single source of data collected may lead to bias from the same source. In our study, the common method variance was not serious. Thus, to improve objectivity and avoid potential bias, we could expand to other data sources for comparison in future study.

Third, the analysis of the model in this current research only considers the individual level. Moreover, recent research called for additional study to explore the impact of intrapreneurship in multiple-level contexts (Gawke et al., 2019). Therefore, future studies should further use multi-level methods to test the results.

Fourth, this research only considered the mediating role of psychological mechanisms. We can extend to other mediators. Moreover, the results only support the partial mediating effect of creative self-efficacy. Future studies can further explore the potential mediating factors (e.g., social capital; Blanka, 2019) to improve the understanding of how and why intrapreneurship affects opportunity recognition.

## Data availability statement

The raw data supporting the conclusions of this article will be made available by the authors, without undue reservation.

## Ethics statement

Ethical review and approval was not required for the study on human participants in accordance with the local legislation and institutional requirements. Written informed consent for participation was not required for this study in accordance with the national legislation and the institutional requirements.

## Author contributions

All authors listed have made a substantial, direct, and intellectual contribution to the work and approved it for publication.

## Conflict of interest

The authors declare that the research was conducted in the absence of any commercial or financial relationships that could be construed as a potential conflict of interest.

## Publisher’s note

All claims expressed in this article are solely those of the authors and do not necessarily represent those of their affiliated organizations, or those of the publisher, the editors and the reviewers. Any product that may be evaluated in this article, or claim that may be made by its manufacturer, is not guaranteed or endorsed by the publisher.
